# Conic tangents based high precision extraction method of concentric circle centers and its application in camera parameters calibration

**DOI:** 10.1038/s41598-021-00300-y

**Published:** 2021-10-19

**Authors:** Fei Hao, Jinjiang Su, Jingjing Shi, Chaohan Zhu, Jiatong Song, Yuntao Hu

**Affiliations:** 1 School of Mechanical Engineering, Nanjing Institute of Technology, Nanjing, China; 2 Nanjing King-Friend Biochemical Pharmaceutical Co., Ltd, Nanjing, China

**Keywords:** Electrical and electronic engineering, Mechanical engineering

## Abstract

A high-precision camera intrinsic parameters calibration method based on concentric circles was proposed. Different from Zhang’s method, its feature points are the centers of concentric circles. First, the collinearity of the projection of the center of concentric circles and the centers of two ellipses which are imaged from the concentric circles was proved. Subsequently, a straight line passing through the center of concentric circles was determined with four tangent lines of concentric circles. Finally, the projection of the center of concentric circles was extracted with the intersection of the straight line and the line determined by the two ellipse centers. Simulation and physical experiments are carried out to analyze the factors affecting the accuracy of circle center coordinate extraction and the results show that the accuracy of the proposed method is higher. On this basis, several key parameters of the calibration target design are determined through simulation experiments and then the calibration target is printed to calibrate a binocular system. The results show that the total reprojection error of the left camera is reduced by 17.66% and that of the right camera is reduced by 21.58% compared with those of Zhang’s method. Therefore, the proposed calibration method has higher accuracy.

## Introduction

Accurate calibration of intrinsic and extrinsic parameters of cameras is a principal problem for machine vision that has been widely explored in the industrial inspection field. It is of the highest importance in camera calibration to find a sufficiently large number of known 3D points in world coordinates and their projections in 2D images^1^. The accuracy of camera calibration is largely dependent on the localization of image points, which is usually evaluated by reprojection errors^2–4^. The reprojection errors represent the error between real image coordinates and reprojected coordinates according to the calibration results.

Most calibration approaches are performed with high-precision or special structure targets that are difficult to manufacture. According to the employed targets, the calibration methods can be divided into 1D methods^5–9^, 2D method^10–26^ and 3D method^2,3,27–31^ and approximate 3D method^32–40^. The targets in 1D methods are usually calibration rods composed of several collinear points. Since they are easily captured by multiple cameras at the same time, this method is often used for multi-camera calibration. However, there are few collinear points, so the calibration accuracy is usually not high. The targets in 2D methods are usually calibration boards with given patterns, such as circles^16–25^, checkerboard^10–15^ or other star-shaped pattern^26^. The targets in 3D methods are usually composed of two or more planes with definite position relationships, which may be helpful to obtain higher calibration accuracy but also leads to the problems of high manufacturing cost and the complex calibration process. Compared with 1D and 3D targets, 2D patterns are easy to design, and there are enough feature points. Therefore, the 2D method is more accurate and simpler.

The chessboard calibration method proposed by Zhang^10^ is one of the most representative 2D calibration methods, which requires four corners and at least three poses of the target. The initial values of camera parameters are obtained through a linear model. Then, an objective function considering nonlinear distortion is established. The maximum likelihood estimation is employed to improve the estimation accuracy of camera parameters. Zhang’s method has high accuracy, and its results are usually used as the ground truth. However, Zhang's method is very time-consuming for multi-camera systems, and its robustness is easily affected by the position of the calibration plate.

To address the above problems, some improved methods of Zhang’s method have been proposed. An adaptive extraction and matching algorithm for checkerboard inner corners was proposed by Qi et al.^12^ to address the problems of manual operation and heavy time consumption in traditional calibration methods. Chung introduced the neural-network model to the checkerboard-based calibration method to compensate for lens distortion^13^. An illumination robust subpixel Harris corner algorithm was proposed, which was employed to improve the checkerboard-based calibration method to achieve high-precision calibration for complicated illumination conditions^14^. Liu et al.^15^ improved the checkerboard-based calibration method by introducing more constraints to the objective function that was established in the 3D coordinate system, such as the adjacent distance constraint, the collinear constraint and the right-angle constraint. Corner detection is a crucial step for Zhang’s method and its improvement, and most of the above improved methods are devoted to improving the accuracy and robustness of corner extraction. However, the corner detection still needs further work.

Because circles are common geometric features in industrial scenes and the circle detection algorithm is more robust than the corner detection algorithm for images with slight defocusing or images captured in low-light environments, circle patterns are typically used to make calibration targets. The camera parameters are calibrated by using the geometric properties and the projective invariances of the circle. A calibration target may contain a single circle or multiple circles. When there are multiple circles in the target, the circles can have various positional relationships, such as separation, concentricity and tangency.

A circular center extraction method based on dual conic geometric characteristics was proposed by Zhao and Liu^21^. And some pairs of end points of diameters can be subsequently determined, which were employed to obtain some pairs of vanishing points of orthogonal directions. Therefore, the intrinsic parameters can be calibrated on the basis of the principle of projective geometry. Liu et al. proposed an intrinsic parameter calibration algorithm by using the conjugate imaginary intersections of two ellipses projected from two coplanar intersecting circles^22^. Zhao et al.^23^ developed a plane target that consists of two concentric circles and straight lines passing through the center of the circles. Based on the invariance of the cross-ratio^41^, the coordinates of the center and vanishing points were obtained, and then the camera intrinsic parameters were calibrated according to the constraints between the image of absolute conic and vanishing points. Last, the lens distortions were corrected by minimizing the objective function established mainly according to the collinearity constraints. Shao et al. proposed a concentric circle-based calibration method^24^, of which the key is to obtain three eigenvectors of the concentric circle projection matrix. The corresponding eigenvectors of two identical eigenvalues represent points on the infinity line, and the other eigenvector is the circle center. Then, the vanishing line of the light plane is obtained from the image of concentric circles. Wang et al. extended Pascal’s theorem to the complex number field. And a pair of conjugate complex points which is the image of a circular point, was calculated accordingly by them. The equations of the camera intrinsic parameters and the conjugate complex points were established on the basis of projective principles, and then the camera intrinsic parameters were directly calibrated^25^. Most of the above methods are based on the principle of projective geometry and use the technique of circular points and the technique of vanishing points and/or vanishing lines. Meng and Hu^19^ were the first to calibrate the camera intrinsic parameters based on the technique of circular points with a hub-and-spoke plane target including six spokes. However, Li et al.^26^ pointed out that Meng and Hu’s method has two major shortcomings. A numerical method should be employed in these methods to solve the conjugate complex points, such as Newton iteration^25^. However, the Newton iterative method is greatly influenced by the initial value, and its calculation burden is relatively large.

Different from the above circle-based calibration method, Zhang et al.^42^ designed a 2.5D calibration target, which is a pyramid containing four coded calibration plates that employ circular features. Zhang’s method does not need complicated numerical calculations to extract the coordinates of the center of the circle. The coordinates of the center of the ellipse, which are equal to the mean values of the coordinates of pixels on the ellipse edge, are directly used as the coordinates of the image of the circle center. However, there is a principle error in using the center of the ellipse to replace the center of the circle, as proven by Ahn et al.^43^. Therefore, Liu et al.^44^ proposed a circle center extraction method, which employs a total of nine points to compensate the principle error mentioned above. However, the error is still large.

A concentric circle center extraction method based on projective properties and geometric constraints is studied in this paper, which only needs linear operation and does not need complex numerical calculation, especially nonlinear iterative operation. Subsequently, a camera intrinsic parameter calibration method based on a concentric circle array is proposed.

The rest of this paper is organized as follows. In “Mathematical model for extracting center coordinates of concentric circles” section introduces a mathematical model for the extraction of the center coordinates of concentric circles. In “Circle center extraction experiment and result analysis” section presents the circle center extraction experiment and result analysis. calibration target design, factor analysis, experimental results, comparisons and discussions are given in “Concentric calibration template and its application” section, and then conclusions are summarized in “Conclusion” section.

## Mathematical model for extracting center coordinates of concentric circles

### Imaging model of circles

The general algebraic equation of an ellipse is

1 $$ \user2{\mathop{X}\limits^{\rightharpoonup} Q\mathop{X}\limits^{\rightharpoonup} }^{T} = 0 $$

where $${\varvec{Q}} = \left[ {\begin{array}{*{20}c} a & {b/2} & {d/2} \\ {b/2} & c & {e/2} \\ {d/2} & {e/2} & f \\ \end{array} } \right]$$ and $$\user2{\mathop{X}\limits^{\rightharpoonup} } = \left[ {\begin{array}{*{20}c} x & y & 1 \\ \end{array} } \right]$$.

Then, the coordinates of the center of the ellipse are

2 $$ x_{ec} = \frac{2cd - be}{{b^{2} - 4ac}}\;{\text{and}}\;y_{ec} = \frac{2ae - bd}{{b^{2} - 4ac}} $$

We establish a world coordinate system whose z-axis is perpendicular to the circle plane and whose origin is at the circle center; then, the circle equation is

3 $$ \user2{\mathop{X}\limits^{\rightharpoonup} C\mathop{X}\limits^{\rightharpoonup} }^{T} = 0 $$

where *r* is the radius of the circle and $${\varvec{C}} = \left[ {\begin{array}{*{20}c} 1 & 0 & 0 \\ 0 & 1 & 0 \\ 0 & 0 & { - r^{2} } \\ \end{array} } \right]$$.

We suppose the homography matrix and its inverse are ***H*** and ***Ω***, respectively.

4 $$ \user2{\Omega = H}^{ - 1} = \left[ {\begin{array}{*{20}c} {h_{11} } & {h_{12} } & {h_{13} } \\ {h_{21} } & {h_{22} } & {h_{23} } \\ {h_{31} } & {h_{32} } & {h_{33} } \\ \end{array} } \right] $$

According to the principle of projective geometry, ***Q*** and ***Ω*** satisfy the following relation.

5 $$ {\varvec{Q}} = \user2{\Omega^{\prime}C\Omega } $$

According to Eq. (5), we can rewrite Eq. (2) as follows.

6 $$ x_{ec} = \frac{{C + r^{2} D}}{{A + r^{2} B}}\;{\text{and}}\;y_{ec} = \frac{{E + r^{2} F}}{{A + r^{2} B}} $$

where *A*, *B*, *C*, *D*, *E* and *F* are six quantities related only to the elements of matrix ***H***.

Two circles with radii of *r*_1_ and *r*_2_ are projected into two ellipses, and the central coordinates of the ellipses are (*x*_1_, *y*_1_) and (*x*_2_, *y*_2_), respectively. Two points determine a straight line on the image plane, and the slope *k* of the straight line is

7 $$ k = \frac{{\left( {BE - AF} \right)}}{{\left( {BC - AD} \right)}} \cdot \frac{{\left( {r_{2}^{2} - r_{1}^{2} } \right)}}{{\left( {r_{2}^{2} - r_{1}^{2} } \right)}} = \frac{BE - AF}{{BC - AD}} $$

This shows that the centers of ellipses projected from the concentric circles are on a straight line that is not related to the radii of the two circles.

If *r*_1_ > *r*_2_ and *r*_2_ = 0, Eq. (6) can be rewritten as

8 $$ x_{c} = {C \mathord{\left/ {\vphantom {C A}} \right. \kern-\nulldelimiterspace} A}\;{\text{and}}\;y_{c} = {E \mathord{\left/ {\vphantom {E A}} \right. \kern-\nulldelimiterspace} A} $$

where (*x*_*c*_, *y*_*c*_) is the pixel coordinate of the circle center.

The slope *k* of the straight line determined by (*x*_1_, *y*_1_) and (*x*_*c*_, *y*_*c*_) is

9 $$ k = \frac{{\left( {BE - AF} \right)}}{{\left( {BC - AD} \right)}} \cdot \frac{{r_{1}^{2} }}{{r_{1}^{2} }} = \frac{BE - AF}{{BC - AD}} $$

This shows that the circle center is on the line determined by (*x*_1_, *y*_1_) and (*x*_2_, *y*_2_), and the pixel coordinates of the circle center can be extracted with the constraint.

### Error of circle center extraction

**Figure 1:**
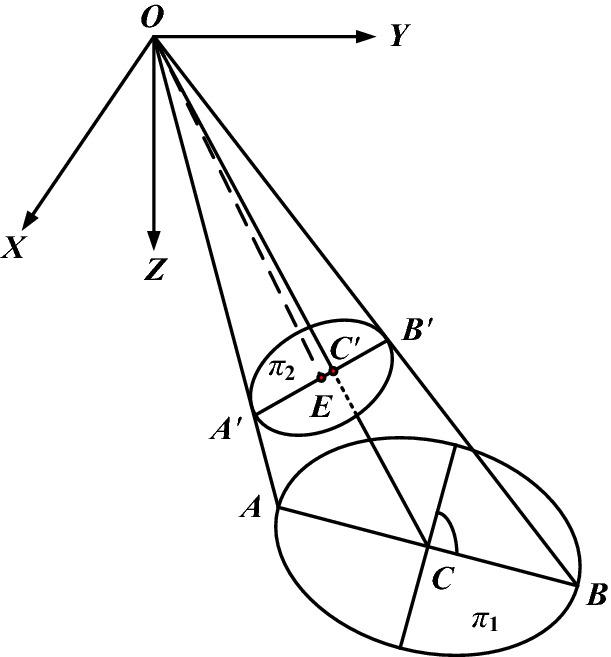
Cause of circle center extraction error.

As shown in Fig. 1, *o*–*xyz* is the camera coordinate system, *π*_1_ is the space plane, and *π*_2_ is the image plane. When the circle on *π*_1_ is projected to *π*_2_, the image is an ellipse. AB is the diameter of the circle, and Aʹ and Bʹ are the images of point A and point B, respectively. Therefore, the midpoint C of AB is the circle center, of which the image is Cʹ, and the midpoint E of AʹBʹ is the ellipse center. When *π*_1_ and *π*_2_ are parallel, E and Cʹ coincide; otherwise, they do not coincide, which leads to error.

**Figure 2:**
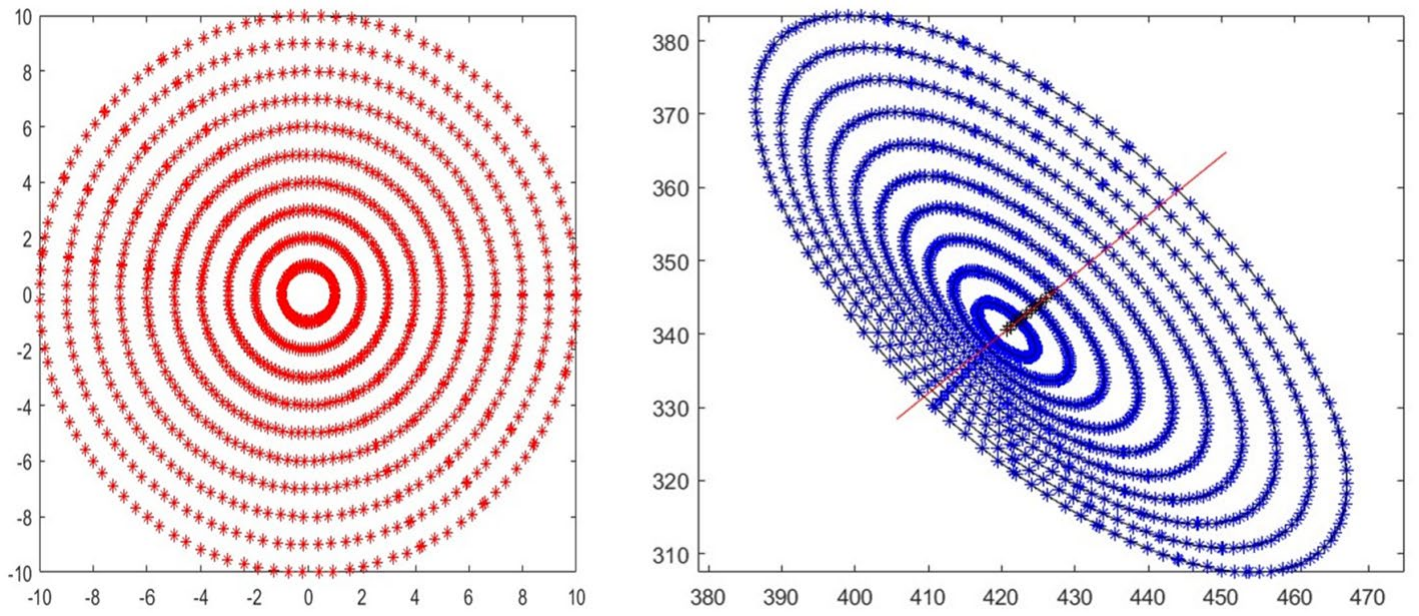
Circles and their projections.

A simulation is carried out to further clarify the error of circle center extraction. The camera intrinsic parameters are *f*_*x*_ = *f*_*y*_ = 3000, *u*_0_ = 320 and *v*_0_ = 240. The camera external parameters are *α* = 20°, *β* = 15°, *γ* = 5°, *x*_0_ = 20 mm, *y*_0_ = 20 mm and *z*_0_ = 600 mm. The radii of the circles range from 1 to 10 mm. A total of 120 points equally spaced on each circle are taken, and each group of 120 points is fitted to obtain 10 ellipses, as shown in Fig. 2.

**Figure 3:**
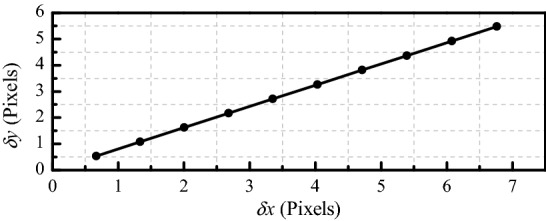
Trend of circle center extraction error.

As shown in Fig. 3, the *x*-axis is *δx* where *δx* = *x*_*c*_ − *x*_*ce*_, and the *y*-axis is *δy*, where *δy* = *y*_*c*_ − *y*_*ce*_. We can confirm that: (1) the ellipse centers are collinear and (2) as the radius increases, the coordinate errors between the ellipse centers and the projection of the circle center also increase.

### Circle center extraction based on projective invariance

**Figure 4:**
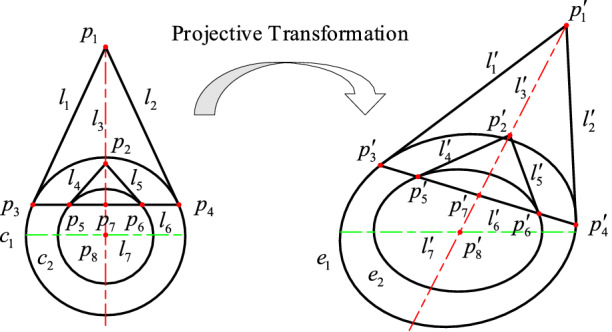
Schematic diagram of the pixel coordinates extraction principle of center projection point.

In projective geometry, a straight line is still a straight line after projection. For a point on a straight line, its projection point is still on the projection line. A straight line is tangent to a circle and a projection line is tangent to the projection of the circle. As shown in Fig. 4, *l*_1_ and *l*_2_ are the tangents of *c*_1_ and their intersection is *p*_1_. *l*_4_ and *l*_5_ are the tangents of *c*_2_ circle and their intersection is *p*_2_. *l*_3_ is a straight line through *p*_1_ and *p*_2_. Line *l*_6_ intersects *c*_1_ at points *p*_3_ and *p*_4_ and *c*_2_ at points *p*_5_ and *p*_6_.

The equations of *c*_1_ and *c*_2_ are

10 $$ \left\{ \begin{gathered} \Phi_{1} \left( {\varvec{x}} \right) \equiv {\varvec{xE}}_{1} {\varvec{x}}^{T} = 0 \hfill \\ \Phi_{2} \left( {\varvec{x}} \right) \equiv {\varvec{xE}}_{2} {\varvec{x}}^{T} = 0 \hfill \\ \end{gathered} \right. $$

If the homogeneous coordinate of $p^{\prime}_{i}$ is $\tilde{\user2{x}}_{i}$ where *i* = 3, 4, 5 and 6. Then the equations of lines $l^{\prime}_{1}$, $l^{\prime}_{2}$, $l^{\prime}_{4}$ and $l^{\prime}_{5}$ are

11 $$ l_{1}^{\prime } = {\varvec{E}}_{1} \cdot \left( {\tilde{\user2{x}}_{3} } \right)^{T} \;{\text{and}}\;l_{2}^{\prime } = {\varvec{E}}_{1} \cdot \left( {\tilde{\user2{x}}_{4} } \right)^{T} $$

12 $$ l_{4}^{\prime } = {\varvec{E}}_{2} \cdot \left( {\tilde{\user2{x}}_{5} } \right)^{T} \;{\text{and}}\;l_{5}^{\prime } = {\varvec{E}}_{2} \cdot \left( {\tilde{\user2{x}}_{6} } \right)^{T} $$

$p^{\prime}_{1}$ is the intersection of $l^{\prime}_{1}$ and $l^{\prime}_{2}$ and $p^{\prime}_{2}$ is the intersection of $l^{\prime}_{4}$ and $l^{\prime}_{5}$. The homogeneous coordinates of $p^{\prime}_{1}$ and $p^{\prime}_{2}$ are

13 $$ p^{\prime}_{1} \equiv \tilde{\user2{x}}_{1} = l^{\prime}_{1} \times l^{\prime}_{2} \;{\text{and}}\;p^{\prime}_{2} \equiv \tilde{\user2{x}}_{2} = l^{\prime}_{4} \times l^{\prime}_{5} $$

The linear equation through $p^{\prime}_{1}$ and $p^{\prime}_{2}$ is

14 $$ l^{\prime}_{3} = \tilde{\user2{x}}_{1} \times \tilde{\user2{x}}_{2} $$

The homogeneous coordinate of the projection of the circle center $p^{\prime}_{8}$ is

15 $$ p^{\prime}_{8} \equiv \tilde{\user2{x}}_{8} = l^{\prime}_{3} \times l^{\prime}_{7} $$

where $l^{\prime}_{7}$ is a straight line through the centers of the ellipses of *e*_1_ and *e*_2_.

Multiple $p^{\prime}_{8}$ can be obtained by involving more $l^{\prime}_{6}$ and then the mean value of the coordinates of multiple $p^{\prime}_{8}$ was obtained which is regarded as the projection of circle center.

## Circle center extraction experiment and result analysis

### Simulation experiment using equal-space points of circles

The intrinsic parameters of the camera remain unchanged. The camera external parameters are *α* = 0 rad, *β* = *π*/3 rad, *γ* = 0 rad, *x*_0_ = 20 mm, *y*_0_ = 20 mm and *z*_0_ = 600 mm. The radius of *c*_2_ ranges from 1 to 10 mm and the radius of *c*_1_ is twice that of *c*_2_. A total of 120 points are selected on each circle at equal intervals, and then 20 ellipses are fitted. The pixel coordinates of the projection of the concentric circle center are extracted by the big-ellipse method, the small-ellipse method, the cross ratio method^41^, the nine-point method^44^ and our method.

**Figure 5:**
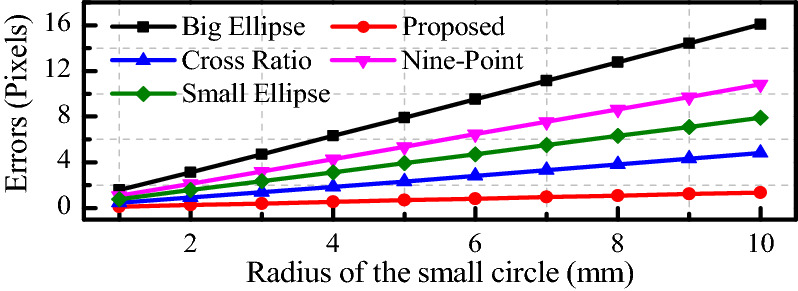
Relationship between coordinate extraction error and radius.

As shown in Fig. 5, the errors of the above methods increase with increasing radius of the circle. The error of our method is the smallest, which is 0.135 pixels, and the error of the cross ratio method is the second smallest, which is 0.453 pixels. The error of the large ellipse method is the largest, which is 4.824 pixels. Compared with the errors of the cross ratio method and the large ellipse method, those of the proposed method are 48.12% and 71.72% smaller, respectively.

**Figure 6:**
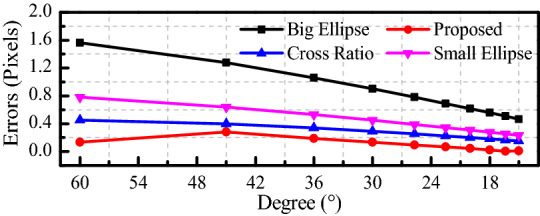
Influence of β on the accuracy.

**Figure 7:**
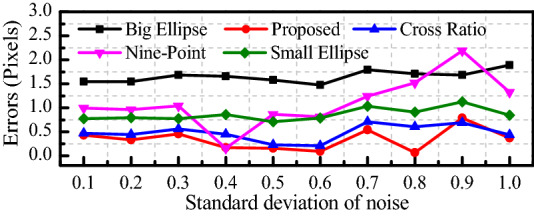
Influence of Gaussian noise on the accuracy.

When the radius of *c*_2_ is 1 mm and that of *c*_1_ is 2 mm, the camera external parameters *α* = *γ* = 0 rad, and *β* ranges from *π*/12 rad to *π*/3 rad. The influence of the camera external parameters on the pixel coordinate extraction accuracy of the circle center is shown in Fig. 6. The influence of Gaussian noise with *μ* = 0 and *σ* = 0.1 ~ 1.0 is shown in Fig. 7.

According to Fig. 6, only the error of the nine-point method increases with *β*. When *β* = π/12 rad, the error of our method is the smallest, which is only 0.007 pixels, while the error of the cross ratio method is 0.168 pixels. The error of our method is approximately 94.64% lower than that of cross ratio method. When *β* = π/3 rad, the error of our method is still the smallest, which is 0.282 pixels, while the error of the cross ratio method is 0.168 pixels. Compared with the cross ratio invariant method, the error of our method is 37.74% smaller.

According to Fig. 7, when *σ* = 0.9, the error of our method is 0.065 pixels larger than that of the cross ratio method. In addition, our method is least affected by noise, and the minimum error of our method is 0.066 pixels, which is 67.60% less than that of the cross ratio method.

### Projective transformation-based simulations

**Figure 8:**
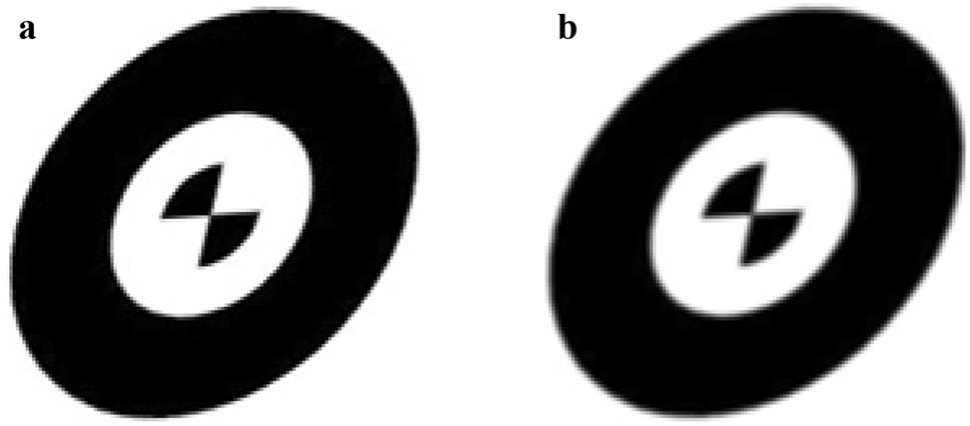
Synthetic images: (**a**) Transformed image and (**b**) smoothed image.

A concentric circle template was drawn by CAD software and then projective geometric transformations are carried out, as shown in Fig. 8a. Then, the transition effects of contour edges are obtained by adding Gaussian noise with *μ* = 0 and *σ* = 0.8 to the transformed image, which is subsequently smoothed, as shown in Fig. 8b.

The radius of *c*_2_ is 1 mm, and that of *c*_1_ is 2 mm. The coordinates extracted by the cross ratio method, the nine-point method and our method are compared with the corner detection results in Table 1.

**Table 1:** Comparison of four coordinate extraction methods.

Images	Corner detection		Cross ratio method		Nine-point method		Our method	
	*u*-axis	*v*-axis	*u*-axis	*v*-axis	*u*-axis	*v*-axis	*u*-axis	*v*-axis
1	419.7276	299.4634	420.0650	299.4356	412.1112	310.3385	419.7086	299.4367
2	424.1969	303.5637	424.5133	303.5479	425.3681	305.7164	423.9503	303.6191
3	414.4020	293.4272	414.7046	293.3752	414.6943	293.5798	414.5639	293.3973
4	371.6168	251.4919	371.8257	251.4602	355.6385	284.8053	371.4036	251.5818
5	405.3264	284.1539	405.6184	284.1070	404.1511	289.0969	405.4819	284.1336
6	413.9713	293.1516	414.3082	293.1516	403.7162	309.5222	413.7421	293.1973
7	419.6232	299.3218	419.9264	299.2687	417.9891	304.3478	419.8437	299.2755
8	418.4337	298.0741	418.7483	298.0644	405.834	292.9833	418.2809	298.1022
9	414.5967	293.7816	414.8942	293.7873	412.3674	290.6162	414.604	293.8136
10	415.1270	294.2009	415.4382	294.1702	412.1112	310.3385	415.5197	294.1622

According to Table 1, the maximum error of the nine point method is 36.9471 pixels, the minimum error is 0.3297 pixels, the average is 11.6565 pixels and the standard deviation is 10.9788 pixels. The maximum and minimum errors of the cross ratio method are 0.3385 pixels and 0.2113 pixels, and the average error and standard deviation are 0.3039 pixels and 0.0355 pixels, respectively. The maximum and minimum errors of our method are 0.3946 pixels and 0.0355 pixels, respectively. The average error and standard deviation are 0.188 pixels and 0.107 pixels, respectively. The error of our method is the smallest, but the cross ratio method is relatively stable.

### Circular imaging experiment and results

**Figure 9:**
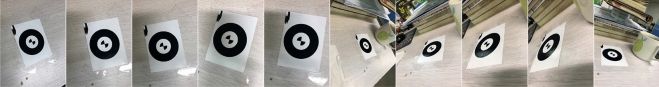
Images of the concentric circle template.

A concentric circle template is printed to further verify the accuracy of coordinate extraction of our method, as shown in Fig. 9.

**Figure 10:**
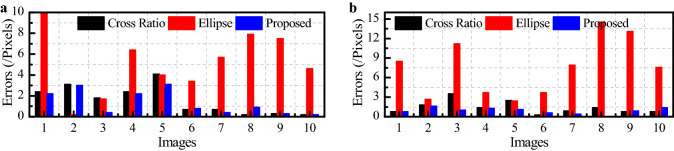
Circle center extraction error of 10 template images: (**a**) *u*-axis and (**b**) *v*-axis.

Because of the large error of the nine-point method, this method is not used. Instead, the corner detection method, cross ratio method, small-ellipse method and our method are studied, and the results are shown in Fig. 10.

According to Fig. 10, compared with the corner method, the maximum error of the small-ellipse method is 28.211 pixels, the minimum error is 2.7074 pixels, the average error is 10.9562 pixels and the standard deviation is 7.5066 pixels. The maximum error of the cross ratio method is 4.8021 pixels, the minimum error is 0.7616 pixels, the average error is 2.2626 pixels and the standard deviation is 1.4764 pixels. The maximum and minimum errors of our method are 3.4 pixels and 0.5657 pixels, and the average error and standard deviation are 1.7491 pixels and 1.0533 pixels, respectively.

Therefore, the accuracy of our method is the highest. Compared with those of the cross ratio method, the maximum error, minimum error, average error and standard deviation of our method are 29.20%, 25.72%, 22.70% and 28.66% less, respectively. Compared with the small ellipse method, the accuracy of our method is higher and the maximum error, minimum error, average error and standard deviation are 87.95%, 79.11%, 84.04% and 85.97% less, respectively.

Our method was compared with a geometric-based algorithm^45^ and an algebraic-based algorithm^46^ to furtherly examine its performance of the feature extraction, both of which are used in newly published articles. The geometric-based algorithm uses the principle that the polar lines intersect at the center of the circles to establish an objective function to extract the center coordinates of the concentric circle. The algebraic-based algorithm obtains the center coordinates of concentric circles by solving the eigenvector of elliptic coefficient matrix. The validation experiments were carried out on a set of ten images and each algorithm was performed five times on images. The mean and standard deviation of the errors of the center coordinate extractions are counted and tabulated in Table 2.

**Table 2:** Results of comparison with a geometric-based and an algebraic-based algorithm.

Images	Statistic	Geometric-based algorithm		Algebraic-based algorithm		Proposed	
		u-axis	v-axis	u-axis	v-axis	u-axis	v-axis
1	μ (pixels)	6.0989	1.2539	1.6429	1.2534	**1.1051**	**1.1832**
	σ (pixels)	1.3851	0.8894	1.3102	**0.4383**	**0.6090**	0.4697
2	μ (pixels)	7.0789	**0.8445**	1.7555	**1.1501**	1.2557	1.1733
	σ (pixels)	0.7615	**0.5766**	0.7635	0.7023	**0.5278**	0.6587
3	μ (pixels)	4.9383	2.1863	1.3734	0.8631	**1.0590**	**0.7738**
	σ (pixels)	1.1855	1.1887	0.9512	**0.7415**	**0.5249**	0.7563
4	μ (pixels)	5.8004	2.8746	1.5443	0.5168	**1.4424**	**0.4776**
	σ (pixels)	0.6357	0.6386	0.6425	0.3579	**0.5823**	**0.3216**
5	μ (pixels)	3.4344	**0.8419**	0.9682	1.1974	**0.5872**	1.1248
	σ (pixels)	1.0239	0.7471	**0.4610**	0.8314	0.7362	**0.7347**
6	μ (pixels)	2.5118	2.5332	**1.1287**	1.2971	1.4023	**1.1896**
	σ (pixels)	1.0418	1.0431	**0.7022**	0.8548	0.7423	**0.7961**
7	μ (pixels)	7.6339	**0.8255**	2.0807	0.8789	**0.7046**	0.8703
	σ (pixels)	0.9254	**0.5146**	0.9294	0.6952	**0.6463**	0.6828
8	μ (pixels)	4.5988	2.4696	1.6673	**0.7947**	**1.5519**	0.9079
	σ (pixels)	**0.6437**	0.6448	0.6443	0.6443	0.9314	**0.6409**
9	μ (pixels)	8.2009	**0.3540**	**0.8205**	0.8041	0.9520	0.8458
	σ (pixels)	0.2638	**0.2408**	0.2624	0.2624	**0.2048**	0.2510
10	μ (pixels)	7.5028	**0.3367**	1.4126	0.4452	**1.0792**	0.5716
	σ (pixels)	0.4152	**0.2022**	**0.4140**	0.4140	0.7361	0.4521

The bold words are the minimums. Every two lines form a group. For the first line of each group, bold words indicate that the mean values of multiple results are close to the true value. For the second line of each group, bold words indicate that the multiple results are close.

According to Table 2, the feature extraction accuracy of the proposal is higher than that of the other two methods. For *u*-coordinates, the mean of circle center extraction errors of the geometry based algorithm, the algebra based algorithm and the proposal are 5.5970, 1.4556 and 1.0957 respectively and the results of the two algorithms are 4.5 and 0.36 larger than that of the proposal; the standard deviation are 2.0573, 0.8268 and 0.6839 pixels respectively and the results of the two algorithms are 1.37 and 0.14 pixels larger than that of the proposal. For *v*-coordinates, the mean are 1.4809, 0.9615 and 0.9424 pixels respectively and the results of the two algorithms are 0.54 and 0.02 pixels larger than that of the proposal; the standard deviation are 1.1301, 0.6650 and 0.6260 pixels respectively and the results of the two algorithms are 0.5 and 0.04 pixels larger than that of the proposal.

Although the accuracy of our method is high, its accuracy and robustness can be further improved by enhancing the ability of edge detection and eliminating outliers to reduce ellipse fitting error.

## Concentric calibration template and its application

### Simulation image generation and experiment

#### Simulation image generation

A rectangular array calibration template consists of *m* rows and *n* columns of concentric circles, and the origin is located in the upper left corner. The center coordinate of the first concentric circle in the upper left corner is (*x*_0_, *y*_0_), and the intervals of the center along the *x*-axis and *y*-axis are Δ*x* and Δ*y*, respectively. Then, the center coordinates of the *i*th column and the *j*th row are

16 $$ x_{i} = x_{0} + \left( {i - 1} \right) \cdot \Delta x\;{\text{and}}\;y_{j} = y_{0} + \left( {j - 1} \right) \cdot \Delta y $$

where $$2 \le i \le n$$ and $$2 \le j \le m$$.

Here, *r*_1_ is the radius of *c*_1_, and *r*_2_ is the radius of *c*_2_. The interval [*r*_2_, *r*_1_] is divided into *m* equal parts, and the interval [0, 2π] is divided into *n* equal parts. According to the pinhole camera model, the noninteger pixel coordinates of projections of all discrete points on circles are obtained on the basis of projective transformation, which are enlarged to create a *K* times magnified image of the template. Then, the magnified image is downsampled with a scaling factor of 1/*K* and smoothed with a mean filter to create the simulation image.

#### Simulation experiment scheme

Here, the influences of image quantities, radius, feature point interval and feature point quantities on the calibration accuracy are analyzed in four groups of simulation experiments with camera parameters *f* = 8 mm, *d*_*x*_ = *d*_*y*_ = 0.00345 mm, *u*_0_ = 1228.3554 pixels, *v*_0_ = 1028.2165 pixels, *s* = 0, *k*_1_ = 0.0823 and *k*_2_ = − 0.02.

1. The influences of circle radii are analyzed where the size of the calibration plate is 160 mm × 160 mm, the interval of feature points is 14 mm, the number of images is 21, the radius ratio of *c*_1_ and *c*_2_ is 2, and the radius of *c*_2_ ranges from 1 to 5 mm.
2. The influences of intervals of feature points are analyzed where the radius of *c*_2_ is 3 mm, the interval of feature points ranges from 4 to 22 mm and other parameters remain unchanged.
3. The influences of the number of images are analyzed where the number of images ranges from 3 to 39 with an interval of 3 and other parameters remain unchanged.
4. The influences of the number of feature points are analyzed where the number of feature points ranges from 44 to 154 with an interval of 22 and other parameters remain unchanged.

#### Result of the simulation experiment

**Figure 11:**
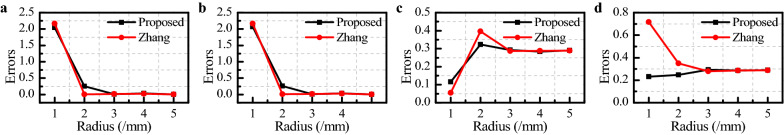
Influences of circle radii: (**a**) *f*_*x*_ error, (**b**) *f*_*y*_ error, (**c**) *u*_0_ error and (**d**) *v*_0_ error.

According to Fig. 11, when the radius of *c*_2_ is larger than 2 mm, the errors of the intrinsic parameters are more stable. The error of *f*_*x*_ decreases with increasing radius, and the minimum error of our method is 0.0047, the minimum error of Zhang’s method is 0.0042; the two are basically the same. The maximum error of our method is 2.045, the maximum error of Zhang’s method is 2.164, and the error of our method is 5.50% less than that of Zhang’s method. The error of *f*_*y*_ also decreases with increasing radius, and the minimum error of our method is 0.0047, the minimum error of Zhang’s method is 0.0055 and the error of our method is 12.96% less than that of Zhang’s method. The maximum error of our method is 2.069, the maximum error of Zhang’s method is 2.166, and the error of our method is 4.48% less than that of Zhang’s method. The minimum error of *u*_0_ is 0.115 pixels and that of Zhang’s method is 0.055 pixels, which is 52.17% smaller. The maximum error of *u*_0_ is 0.324 pixels and that of Zhang’s method is 0.396 pixels, which is 18.18% larger. The minimum error of our method *v*_0_ is 0.246 pixels, the minimum error of Zhang’s method is 0.279 pixels, and the error of our method is 11.83% less than that of Zhang’s method. The maximum error of our method *v*_0_ is 0.294 pixels, the maximum error of Zhang’s method is 0.716 pixels, and the error of our method is 0.422 pixels less than that of Zhang’s method.

**Figure 12:**
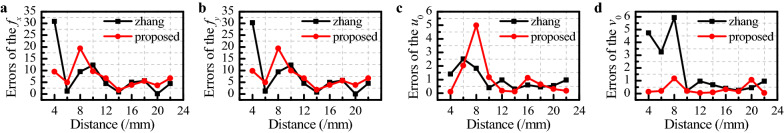
Influences of intervals: (**a**) *f*_*x*_ error, (**b**) *f*_*y*_ error, (**c**) *u*_0_ error and (**d**) *v*_0_ error.

According to Fig. 12, with the increase in the intervals between the feature points, the errors decrease. The minimum error of *f*_*x*_ of our method is 1.738 and the minimum error of Zhang’s method is 0.094. The maximum error of our method is 19.336 and the maximum error of Zhang’s method is 30.882, which is 37.39% larger. The minimum error of *f*_*y*_ of our method is 1.783 and the minimum error of Zhang’s method is 0.0069. The maximum error of our method is 19.374 and the maximum error of Zhang’s method is 30.262, which is 35.98% larger. The minimum error of *u*_0_ of our method is 0.113 pixels, and the minimum error of Zhang’s method is 0.284 pixels, so the minimum error of our method is half that of Zhang’s method. The maximum error of our method is 4.996 pixels, and the maximum error of Zhang’s method is 2.519 pixels, so the error of our method is larger than that of Zhang’s method. The minimum error of *v*_0_ of our method is 0.048 pixels, and the minimum error of Zhang’s method is 0.217 pixels, so the error of our method is 77.88% less than that of Zhang’s method. The maximum error of our method is 1.177 pixels, and the maximum error of Zhang’s method is 5.939 pixels, so the error of our method is 4.762 pixels less than that of Zhang’s method. Generally, when the interval is more than 14 mm, the error of camera intrinsic parameters is small. Therefore, the interval of 14 mm may be appropriate.

**Figure 13:**
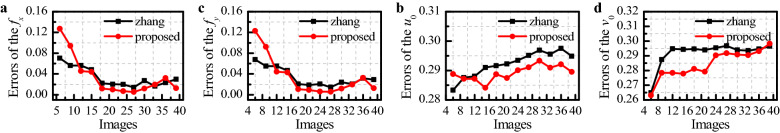
Influences of the number of images: (**a**) *f*_*x*_ error, (**b**) *f*_*y*_ error, (**c**) *u*_0_ error and (**d**) *v*_0_ error.

According to Fig. 13, when the number of images is 12 to 30, the error of our method is less than that of Zhang’s method. As the number of images increases, the error in *f*_*x*_ decreases. The minimum error of our method is 0.0052, and the minimum error of Zhang’s method is 0.011, so the minimum error of our method is approximately half that of Zhang’s method. The maximum error of our method is 0.082, and the maximum error of Zhang’s method is 0.101, so the error of our method is 18.81% less. The minimum error of *f*_*y*_ of our method is 0.0044, and the minimum error of Zhang’s method is 0.0107, so the error of our method is 58.88% less than that of Zhang’s method. The maximum error of our method is 0.087, and the maximum error of Zhang’s method is 0.103, so the error of our method is 15.53% less than that of Zhang’s method, and the error is small when the number of images is between 18 and 30. The minimum error of *u*_0_ of our method is 0.292 pixels, and the minimum error of Zhang’s method is 0.287 pixels, so the error of our method is 1.74% less than that of Zhang method. The maximum error of our method is 0.322 pixels, and the maximum error of Zhang’s method is 0.311 pixels, so the results of our method are 3.42% larger than that of Zhang’s method. The minimum error of *v*_0_ of our method is 0.285 pixels, and the minimum error of Zhang’s method is 0.265 pixels, so the result of the method is 7.02% larger than that of Zhang’s method. The maximum error of our method is 0.312 pixels, and the maximum error of Zhang’s method is 0.302 pixels, so the result of our method is 3.21% larger than that of Zhang’s method. Generally, when the number of images is between 18 and 27, the calibration error is small. Therefore, 21 images may be appropriate.

**Figure 14:**
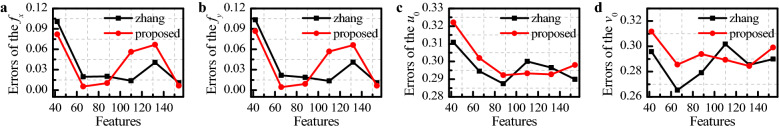
Influences of the number of feature points: (**a**) *f*_*x*_ error, (**b**) *f*_*y*_ error, (**c**) *u*_0_ error and (**d**) *v*_0_ error.

According to Fig. 14, when the number of feature points is 88, the calibration error is small. The error of *f*_*x*_ of our method is 0.01 and is 0.02 for Zhang’s method. The error of our method is approximately half that of Zhang’s method. The error in *f*_*y*_ of our method is 0.0092, and that of Zhang’s method is 0.0217, which is 57.6% larger. The error in *u*_0_ of our method is 0.292 pixels and that of Zhang’s method is 0.287 pixels. The result of our method is 1.7% larger that of Zhang’s method. The error in *v*_0_ of our method is 0.294 pixels and 0.279 pixels for Zhang’s method. The result of our method is 5.1% larger that of Zhang’s method. Although the error in *u*_0_ and *v*_0_ is slightly larger than that of Zhang’s method, the maximum difference is less than 0.015 pixels. Therefore, we carefully infer that when the radius is 3 mm, the number of images is 21, the interval of feature points is 14 mm and the number of feature points is 88, a better calibration result is achieved.

#### Reprojection error

Table 3 tabulates the reprojection errors of the two methods in the pixel coordinate system. According to Table 3, the reprojection error of our method along the *u*-axis is 0.26% less than that of Zhang’s method, while the projection error of our method along the *v*-axis is 16.41% less than that of Zhang’s method. This means that the calibration accuracy of our method is slightly higher than that of Zhang’s method.

**Table 3:** Reprojection errors of two methods.

Method	*u*-axis (pixels)	*v*-axis (pixels)
Proposed	0.06948	0.08168
Zhang	0.06966	0.10757

### Binocular system calibration experiment

**Figure 15:**
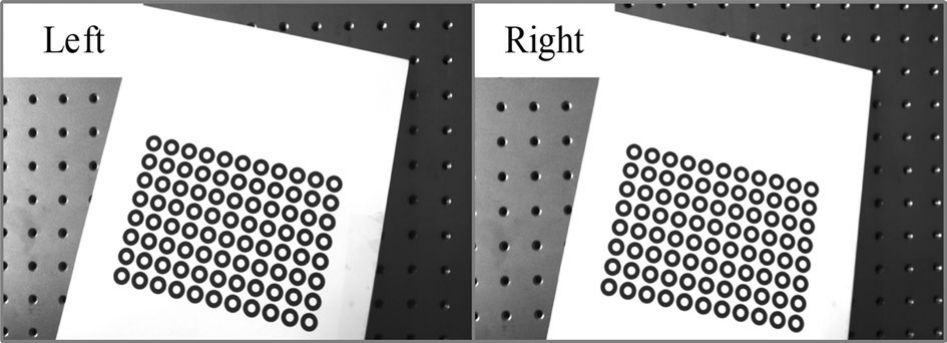
Images of the calibration board.

Based on the simulation results, the calibration board is made to calibrate a binocular vision system with cameras of MV-EM510M/C. The images of the calibration board collected by the left and right cameras are shown in Fig. 15.

The calibration results of camera intrinsic parameters including distortion parameters, are shown in Table 4. The results of our method are basically consistent with those of Zhang’s method, and our method is found to have high stability and reliability. Although the two cameras are of the same model, the intrinsic parameters are not the same. It is necessary to calibrate the parameters separately to ensure the measurement accuracy of the binocular system.

**Table 4:** Calibration results of intrinsic parameters of the left and right cameras.

Group	Camera	*f*_*x*_/mm	*f*_*y*_/mm	*u*_0_/pixel	*v*_0_/pixel	*k*_1_	*k*_2_
1	Left	2298.8167	2294.2042	1222.4215	1027.7098	− 0.1250	0.3544
	Right	2328.6392	2322.7206	1235.9632	1020.8740	− 0.1261	0.2812
2	Left	2289.6233	2301.1630	1228.8542	1020.6600	− 0.1241	0.3460
	Right	2316.3532	2316.3586	1232.3203	1028.8509	− 0.1332	0.3087

The reprojection errors of the two groups of experiments are calculated as shown in Table 5. In general, the proposed method achieves lower reprojection error. The minimum reprojection error for the *u*-axis is 0.17913 pixels, and that for the *v*-axis is 0.23204 pixels. However, the left camera reprojection error of Zhang's method for *u*-axis is 2.55% lower than that of our method in the second experiment. The total projection errors of the left camera and the right camera are also calculated. The total reprojection error of the left camera of our method is 0.4037 pixels, and that of the Zhang method is 0.4903 pixels, which is 17.66% less than that of the Zhang’s method. The total reprojection error of the right camera is 0.3136 pixels, and that of the Zhang method is 0.3999 Compared with Zhang’s method, the proposed method has a 21.58% smaller value, which shows that the overall accuracy of the proposed method is higher, but there are also a small number of problems of large deviation and discrete results, which may be caused by two reasons.

1. There are inevitable errors in the manufacture of printed calibration plates, such as the flatness error of the plate and the roundness error of the concentric circle.
2. The uniform illumination condition is difficult to guarantee and therefore the consistency of image quality is reduced, which leads to the fluctuation of ellipse fitting error and affects the accuracy of circle center extraction.

**Table 5:** Reprojection errors of two groups of experiments.

Group	Camera	*u*-axis		*v*-axis	
		Proposed	Zhang	Proposed	Zhang
1	Left	0.28191	0.28904	0.29720	0.38997
	Right	0.18073	0.28446	0.23204	0.38196
2	Left	0.30122	0.29373	0.31205	0.38087
	Right	0.17913	0.25741	0.23267	0.32524

## Conclusion

In this paper, a camera intrinsic parameter calibration method based on a concentric circle array was proposed without corner extraction. The main work included the following: a high precision extraction method of concentric circle centers based on projective characteristics and geometric constraints was proposed, methods of simulation image creation were explored, and corresponding simulation and physical experiments were carried out. The experimental results showed that the total reprojection errors of the left camera and the right camera were reduced by 17.66% and 21.58% compared with Zhang’s method, respectively. Therefore, the proposed calibration method has high accuracy. The calibration accuracy is expected to be further improved by improving the accuracy of edge detection and achieving the accuracy design of the calibration target.

## Data Availability

All data generated or analysed during this study are included in this published article.
